# Utilization of outpatient medical care in Germany

**DOI:** 10.17886/RKI-GBE-2017-127

**Published:** 2017-12-13

**Authors:** Franziska Prütz, Alexander Rommel

**Affiliations:** Robert Koch Institute, Department of Epidemiology and Health Monitoring, Berlin

**Keywords:** OUTPATIENT CARE, ADULTS, HEALTH SERVICES RESEARCH, HEALTH MONITORING, GERMANY

## Abstract

Outpatient care in Germany is mainly provided in physicians’ private practices. Data from health surveys enable to analyse the utilization of outpatient services from the patient’s perspective, and to identify associations with social determinants and other influencing factors. As part of the GEDA 2014/2015-EHIS study, data was collected for the indicator ‘utilization of outpatient medical care in the last 12 months’. The analysis found that 90.9% of women aged 18 years and over and 84.1% of men had used outpatient medical services within the last 12 months. The utilization of outpatient medical care increases with age: about 94% of people aged 65 or over were provided with outpatient medical treatment in the last 12 months. There were significant differences in utilization between younger men and women, which balance out with age. No differences were identified in relation to educational level or federal state.

## Introduction

Outpatient care refers to all forms of health care provided outside of hospitals. Outpatient medical care is the largest area in this field. In Germany, the vast majority of outpatient medical care is provided by office-based general practitioners (GPs) and specialists working in private practice. Doctors’ practices are usually the first port of call within the professional health care system for people with health problems. In Germany, general practitioners and specialists diagnose health problems and identify the need for treatment, carry out examinations and treatments and, if necessary, arrange further health and social services [1]. In 2015, EUR 172.3 billion was paid for outpatient medical treatment in Germany. This corresponds to about half of the total expenditure on health. Outpatient health care is provided not only by medical and dental practices but also by practices of other health care practitioners, by pharmacies, retailers and other providers of medical goods and by providers of home health care services. Health care expenditure provided in physicians’ practices amounted to EUR 51.6 billion in 2015 [2].

The utilization of outpatient health care provided in medical practices can be analysed using data from official statistics, service providers, and statutory health insurance as well as from population-representative health surveys. However, since the invoicing modalities changed in 2008, accounting data can only be used to calculate the number of cases per quarter that were treated at a specific doctor’s practice, but no longer the contact frequencies during a quarter. In addition, accounting data from health insurances often refer only to specific groups of insured people [3, 4]. In contrast, survey data enable to analyse the utilization of outpatient medical care from the patient’s perspective and to identify associations with social determinants and other influencing factors [5]. Thus, these data is also of great interest for needs-based planning in outpatient care. Current data on the utilization of outpatient medical care are available from the German Health Update (GEDA 2014/2015-EHIS) study. The study is part of the Health Monitoring framework at the Robert Koch Institute (RKI) and the European health monitoring system; the questions asked are part of the European Health Interview Survey (EHIS) and were integrated into the GEDA study.

**GEDA 2014/2015-EHIS**

**Data holder:** Robert Koch Institute

**Aims:** To provide reliable information about the population’s health status, health-related behaviour and health care in Germany, with the possibility of a European comparison

**Method:** Questionnaires completed on paper or online

**Population:** People aged 18 years and above with permanent residency in Germany

**Sampling:** Registry office sample; randomly selected individuals from 301 communities in Germany were invited to participate

**Participants:** 24,016 people (13,144 women; 10,872 men)

**Response rate:** 26.9%

**Study period:** November 2014 - July 2015

**Data protection:** This study was undertaken in strict accordance with the data protection regulations set out in the German Federal Data Protection Act and was approved by the German Federal Commissioner for Data Protection and Freedom of Information. Participation in the study was voluntary. The participants were fully informed about the study’s aims and content, and about data protection. All participants provided written informed consent.

More information in German is available at www.geda-studie.de

## Indicator

In the GEDA 2014/2015-EHIS study, data for the indicator ‘utilization of outpatient medical care in the last 12 months’ was collected using a self-reporting questionnaire completed by the participants on paper or online. The participants were asked two questions. First, ‘When was the last time you consulted a general practitioner or family doctor on your own behalf? Please include visits to the doctor’s practice, home visits and consultations by phone’; second, ‘The next question is about outpatient examinations and treatment undertaken by specialists. It does not refer to visits to the dentist. When was the last time you consulted a medical specialist on your own behalf?’ The following answers were possible: ‘Less than 12 months ago’, ‘12 months ago or longer’ and ‘Never’. The answers provided to these two questions were aggregated for the analysis of the utilization of outpatient medical care. Participants were considered to have used outpatient medical treatment if they answered ‘Less than 12 months ago’ to at least one of the questions.

The following analyses are based on data from 23,921 participants aged 18 or above (13,092 women; 10,829 men) with valid data on the utilization of outpatient medical care. Calculations for the study were carried out using a weighting factor that corrected the sample for deviations from the structure of the German population (as of 31 December 2014) regarding gender, age, district type and level of education. The district type reflects the degree of urbanisation and corresponds to the regional distribution in Germany. The International Standard Classification of Education (ISCED) was used to classify the participants’ educational and occupational qualifications [6]. Differences between groups are interpreted as statistically significant when the respective confidence intervals do not overlap.

A detailed description of the methodology used for GEDA 2014/2015-EHIS can be found in Lange et al. 2017 [7] as well as in the article German Health Update: New data for Germany and Europe in issue 1/2017 of the Journal of Health Monitoring.

## Results and discussion

Most adults – almost 9 out of 10 – use outpatient medical care at least once a year. Women (90.9%) have a significantly higher rate of utilization than men (84.1%, Table 1).

A detailed analysis stratified by age shows an increase of utilization from the 45-to-64 year age group. Differences between women and men are most evident at a young age: among 18- to 29-year-olds, 90.4% of women and 78.4% of men used outpatient medical care in the 12 months that preceded the study. The same applies to 87.8% and 77.6% of 30- to 45-year-old women and men, respectively. The differences in utilization between women and men balance out with age, with no differences observed among people aged 65 or above (94.0% of women and 93.7% of men sought medical assistance at least once in the 12 months that preceded the study). There are no significant differences in utilization with regard to educational level, or according to federal state compared with the national average.

GEDA 2012 found similar results to those identified by GEDA 2014/2015-EHIS: 90.5% of women and 83.6% of men reported that they had visited a doctor at least once in the last 12 months [8]. However, in contrast to GEDA 2014/2015-EHIS, where two questions were combined for the analysis, in GEDA 2012 and the earlier GEDA waves data on utilization of outpatient medical care were collected using only one question. In addition, the survey mode used for GEDA 2014/2015-EHIS was different to that used in earlier studies: it changed from a telephone to a paper or online survey. As such, comparability of the data is limited, and reliable statements about time trends are not possible. The GEDA waves that were conducted between 2009 and 2012 identified a statistically significant increase in the utilization of outpatient medical treatment within the 12 months preceding the studies (it rose from 84.9% to 87.1%) [8]. Analyses of accounting data from the statutory health insurer BARMER show a slightly higher share of people with health insurance visiting physicians’ practices in 2014 and 2015 (around 93%) [9, 10]. In the case of self-reported data on the utilization of medical care, there is the possibility of recall bias, or errors may occur, because very old or severely ill people cannot participate in surveys [4, 11]. In particular, when people are older, they seem to underestimate their visits to physicians’ practices [12]. However, this problem applies more to the number of physician contacts than to the question of whether a physician is consulted at all. Moreover, recall bias is more likely to occur when data is collected on periods stretching to more than 12 months in the past [13]. GEDA 2014/2015-EHIS did not collect data on the reason for seeking medical care.

Earlier GEDA studies have also shown that especially in a younger age, women visit medical practices more frequently than men [8, 14, 15]. To a large extent, this is likely to be due to visits to gynaecological practices (e.g. for cervical cancer screening, consultations about contraception or care during pregnancy). In addition, women report a greater degree of sensitivity toward issues concerning the body and health in general and are also more willing to accept medical help [16]. In contrast, men often only make use of medical services to the same extent as women once they are affected by disease [17]. This is also reflected in the finding that there is no significant difference in the utilization of outpatient medical treatment provided in physicians’ practices among adults aged 65 or above [12] – a finding which is also a result of GEDA 2014/2015-EHIS.

In terms of educational level, GEDA 2014/2015-EHIS found no differences in the utilization of outpatient medical care; this confirms the findings of GEDA 2012. However, a more detailed analysis of the utilization of outpatient medical treatment provided by GPs, family doctors and specialists reveal differences according to socio-economic status [18-20]: people with low socio-economic status are more likely to use services provided by GPs, whereas people with high socio-economic status have a higher utilization of specialists. The data collected for GEDA 2014/2015-EHIS can be used for further analyses of the utilization of medical treatment provided by GPs/family doctors and specialists, including the frequency of consultation and regional differences. Such analyses should not only differentiate between rural and urban regions, but also take into account the ratio of (specialist) doctors to the population and the accessibility of medical practices [21].

The indicator ‘utilization of outpatient medical care in the last 12 months’ maps the utilization of outpatient medical services from the patient’s perspective. As such, it complements the data from official statistics, service providers and statutory health insurance, such as for needs- and demand-based planning of outpatient services. Together with other contributions to this issue (Fact sheets on the utilization of hospital treatment, the utilization of physiotherapy, the use of medically prescribed medicines and self-medication, and the Focus on the utilization of psychotherapeutic and psychiatric care), this Fact sheet provides an overview of essential aspects of the utilization of health care by adults in Germany.

## Key statements

- Nearly 90% of adults use outpatient medical care every year.
- In particular, women under the age of 45 visit doctors’ practices more frequently than men; to a large extent, this is likely to be due to visits to gynaecologists.
- Utilization increases with age: about 94% of people aged 65 years or above received outpatient medical care in the last 12 months.
- No differences were identified in the utilization of outpatient medical care according to education.

## Figures and Tables

**Table 1:** 12-month prevalence of utilization of outpatient medical care according to gender, age and educational level (n=13,092 women; n=10,829 men) Source: GEDA 2014/2015-EHIS

Women	%	(95% CI)	Men	%	(95% CI)
**Women (total)**	**90.9**	**(90.2-91.6)**	**Men (total)**	**84.1**	**(83.1-85.0)**
**18-29 Years**	90.4	(88.7-91.9)	**18-29 Years**	78.4	(75.7-80.8)
Low education	90.9	(86.2-94.1)	Low education	79.3	(73.4-84.2)
Medium education	90.2	(88.0-92.1)	Medium education	79.1	(75.7-82.1)
High education	90.4	(87.0-92.9)	High education	72.7	(66.7-78.0)
**30-44 Years**	87.8	(86.2-89.3)	**30-44 Years**	77.6	(75.2-79.9)
Low education	87.1	(81.6-91.1)	Low education	77.1	(68.8-83.6)
Medium education	87.1	(85.0-89.0)	Medium education	79.1	(75.7-82.2)
High education	90.1	(87.7-92.1)	High education	75.3	(71.9-78.4)
**45-64 Years**	90.8	(89.7-91.8)	**45-64 Years**	85.0	(83.6-86.4)
Low education	90.3	(87.3-92.6)	Low education	84.9	(80.5-88.4)
Medium education	90.6	(89.3-91.8)	Medium education	86.3	(84.1-88.2)
High education	91.7	(90.1-93.0)	High education	82.7	(80.7-84.6)
**≥65 Years**	94.0	(92.8-95.0)	**≥65 Years**	93.7	(92.5-94.6)
Low education	94.1	(92.3-95.5)	Low education	93.1	(89.9-95.3)
Medium education	93.7	(91.9-95.2)	Medium education	93.9	(92.2-95.3)
High education	94.7	(92.1-96.4)	High education	93.5	(91.7-94.9)
**Total (women and men)**	**87.6**	**(87.0-88.2)**	**Total (women and men)**	**87.6**	**(87.0-88.2)**

CI=confidence interval
